# Bringing Climate Action to Clinics: Integrating Green Policies in Health Care

**DOI:** 10.31729/jnma.9200

**Published:** 2025-08-31

**Authors:** Aishana Joshi

**Affiliations:** 1 Editor, Journal of Nepal Medical Association, Kathmandu, Nepal

Climate change presents a fundamental threat to human health and communities worldwide. As global temperatures continue to rise and extreme weather events become more frequent, the health impacts of climate change have increased substantially in recent years.^1,2^ Climate change is directly contributing to humanitarian emergencies ranging from heatwaves, wildfires, droughts, floods, tropical storms and hurricanes to the spread of infectious diseases and worsening of chronic conditions.^3^ According to the World Health Organization (WHO), between 2030 and 2050, climate change is projected to cause approximately 250,000 additional deaths per year from undernutrition, malaria, diarrhoeal diseases and heat stress.^4^ The climate crisis is no longer a distant threat; it is an urgent and pressing public health emergency that demands action from all sectors of society, including health care. In this context, the health sector must evolve not only to respond to climate related health issues but also serve as a driving force in tackling the root cause of climate crisis. Green policies in health care offer a vital pathway towards this goal, and it is time to integrate climate action directly into the frontline of our health systems.

Health care is both a contributor and victim of climate change. The sector is responsible for approximately 4-5% of global carbon emissions primarily from the energy-intensive operations of medical facilities, pharmaceutical production and waste generation.^5^ At the same time, climate change affects every aspect of health systems including supply-chain disruption, infrastructure damage, workforce shortages and an escalating burden of health issues. The health facilities are witnessing a rising incidence of respiratory illnesses, heat related conditions, vector-borne diseases and mental health disorders driven by climate stressors.^6-8^ In response to this dual challenge, health systems around the world must adopt green policies that not only reduce the environmental footprint but also prepare for the inevitable health threats of climate change. Climate action should be integrated as a core aspect of healthcare delivery at every level.

In May 2025, the World Health Assembly adopted the Global Action Plan on Climate Change and Health, articulating a global roadmap to strengthen health systems resilience and mitigate the profound impacts of climate change.^9^ Health care settings can begin by implementing energy-efficient practices, such as adopting renewable energy sources like solar and wind, solar powered medical equipment and smart building technologies. A transition towards green building standards not only reduces carbon footprints but also leads to long term cost reductions allowing health care institutions to reallocate resources for better patient care. Green policies must also encompass the procurement of products by health care facilities, ensuring integration of sustainability throughout the supply chain. Health care professionals must be equipped with knowledge and skills to tackle climate-related health issues, design sustainable health care systems and influence policy decisions. Digital health solutions and telemedicine play a crucial role in reducing carbon emissions associated with transportation and in-person consultations.^10^ Health facilities can adopt practices that reduce waste generation by encouraging use of eco-friendly medical equipment and improving waste segregation and disposal methods. Green policies and sustainable health care practices improve air quality, reduce exposure to environmental pollutants and promote mental well-being as individuals benefit from positive effects of greener spaces.

Climate change is a complex and multifaceted challenge, yet there are practical, actionable steps that health systems can implement to safeguard global public health. The adoption of green policies in health care significantly benefits the environment as well as enhances patient health. By aligning health care practices with green policies, the sector can address immediate health needs while actively contributing to curb the global climate crisis. This is a call to action for the medical community to recognize and actively respond to climate-health challenges through proactive solutions that prioritize both public and planetary health.
